# Exosome-transmitted miR-3124-5p promotes cholangiocarcinoma development *via* targeting GDF11

**DOI:** 10.3389/fonc.2022.936507

**Published:** 2022-08-01

**Authors:** Huijie Gao, Zhaobin He, Chao Gao, Naiqing Liu, Zhaoyang Zhang, Weibo Niu, Jun Niu, Cheng Peng

**Affiliations:** ^1^ Department of Hepatobiliary Surgery, General Surgery, Qilu Hospital, Cheeloo College of Medicine, Shandong University, Jinan, China; ^2^ The Institute of Laparoscopic Minimally Invasive Surgery of Shandong University, Jinan, China; ^3^ Department of General Surgery, Linyi Central Hospital, Linyi, China; ^4^ Department of Emergency Medicine, Qilu Hospital, Cheeloo College of Medicine, Shandong University, Jinan, China

**Keywords:** cholangiocarcinoma, exosome, miRNA, miR-3124-5p, growth differentiation factor 11

## Abstract

**Objective:**

Cholangiocarcinoma (CHOL) is a deadly cancer worldwide with limited available therapies. The aim of this study was to investigate key exosomal miRNAs and their functions in CHOL development.

**Methods:**

Serum exosomes were isolated from patients with CHOL and healthy controls, followed by miRNA sequencing for identifying differentially expressed miRNAs (DEMs) and their functions. Then, the expression of key DEMs was experimentally validated in exosomes from clinical CHOL patients and CHOL cells. The effects of overexpression of key DEMs on CHOL cell migration and proliferation were investigated. A key exosomal DEM miR-3124-5p was identified. The effects of overexpression or knockdown of exosomal miR-3124-5p on the proliferation, migration, and angiogenesis of human umbilical vein endothelial cells (HUVECs) were investigated. Moreover, the function of exosomal miR-3124-5p on tumor growth *in vivo* was explored.

**Results:**

A total of 632 exosomal DEMs were identified between CHOL and control samples. Target genes of DEMs were significantly enriched in pathways, such as the p53 signaling pathway. miR-3124-5p was upregulated in serum exosomes from CHOL patients and exosomes from CHOL cells, and overexpression of miR-3124-5p promoted RBE cell migration and viability. Moreover, overexpression of exosomal miR-3124-5p promoted the proliferation, migration, and angiogenesis of HUVECs, while knockdown of miR-3124-5p had the opposite effect. miR-3124-5p could target growth differentiation factor 11 (GDF11) and downregulate GDF11 expression. Furthermore, exosomal miR-3124-5p promoted tumor growth *in vivo*.

**Conclusions:**

Our findings revealed that exosome-encapsulated miR-3124-5p promoted the malignant progression of CHOL by targeting GDF11. Exosomal miR-3124-5p and GDF11 could be promising biomarkers or therapeutic targets for CHOL.

## Introduction

Cholangiocarcinoma (CHOL) is a highly lethal malignancy of the bile duct epithelium, accounting for 3% of all gastrointestinal malignant tumors (1). According to anatomical origin, CHOL can be classified into intrahepatic, hilar, and extrahepatic groups (2). Due to the strong occult invasion, most CHOL patients are diagnosed at an advanced stage, which leads to poor prognosis, with only 7%–20% of patients reaching 5-year survival (3, 4), and severely limits treatment options (5). Even patients undergoing potentially curative surgical resection have high rates of recurrence and early local or distant metastases (6, 7). Therefore, understanding the molecular mechanisms underlying CHOL development will help in the identification of novel diagnostic biomarkers and development of therapeutic strategies.

Exosomes are endosome-derived nanosized extracellular vesicles ranging from 40 to 160 nm in diameter (8). They are broadly distributed in multiple body fluids, such as blood, urine, cerebrospinal fluid, and bile, and have good stability (9). Accumulating studies have revealed that exosomes play an essential role in cell–cell communication in the tumor microenvironment, and thus contribute to tumorigenesis and development of a variety of cancers (10, 11). In addition, exosomes contain a wide range of components, such as proteins, lipids, nucleic acids, mRNAs, and microRNAs (miRNAs), which are clinically important as diagnostic biomarkers, therapeutic targets, and anti-cancer drug delivery vehicles (12).

miRNAs represent a group of small regulatory RNA with lengths oscillating 20–22 nucleotides each, which are implicated in a wide range of human diseases including cancers via regulating target gene expression either through inhibiting translation or through degrading target mRNAs (13, 14). Increasing lines of evidence have shown that high levels of miRNAs are contained in exosomes, and exosomal miRNAs exert significant function in occurrence and development of cancers (15). Exosomal miR-23a-3p plays a key role in CHOL development by interaction with Dynamin3 (16). Serum exosomal miR-200 family is recognized as diagnostic biomarkers for CHOL (17). However, the key exosomal miRNAs as well as their role and regulatory mechanism in CHOL have not been fully explored.

In the present study, we conducted miRNA sequencing to identify differentially expressed miRNAs (DEMs) in the serum exosomes of patients with CHOL and analyzed their functions. We then investigated the function and regulatory mechanism of a key exosomal DEM miR-3124-5p, which was upregulated in serum exosomes of patients with CHOL, in exosomes of CHOL cells, and human umbilical vein endothelial cells (HUVECs). Moreover, we explored the target gene of miR-3124-5p to elucidate the possible mechanism of miR-3124-5p in regulating CHOL development. Our findings will provide a theoretical basis for developing novel therapeutic strategies for CHOL patients.

## Materials and methods

### Patients and samples

Blood samples were collected from 13 healthy individuals and 13 patients with CHOL. After centrifugation at 5,000 rpm for 10 min at 4°C, the serum was collected and stored at −80°C until further use. This study was approved by the Ethics Committee of Qilu Hospital of Shandong University and documented informed consent was obtained from each subject.

### Isolation and characterization of exosomes

Serum exosomes were isolated by the hypervelocity centrifugation method. In detail, the collected serum was centrifuged at 2,000 ×g for 15 min. The supernatant was then centrifuged at 10,000 ×g for 30 min to remove impurities. The supernatant was further ultracentrifuged at 120,000 ×g for 70 min to collect the pellets. The pellets were resuspended in PBS and subjected to ultracentrifugation at 120,000 ×g for 70 min. The obtained pellets were exosomes that were resuspended with PBS and stored at −80°C.

The isolated exosomes were then observed by transmission electron microscopy (TEM), and the size of exosomes was measured by nanoparticle tracking analysis (NTA) using the ZetaView PMX 110 analyzer (Particle Metrix, Meerbusch, Germany). Moreover, the protein levels of the exosomal markers, including HSP70, CD81, and tumor susceptibility gene 101 (TSG101), were determined using Western blot assay.

### Exosomal RNA isolation, miRNA library construction, and miRNA sequencing

Total RNA was extracted from serum exosomes from three healthy individuals and three patients with CHOL (two intrahepatic and one extrahepatic) using Trizol (Invitrogen, Carlsbad, CA, USA) followed by the protocols provided by the manufacturer. Subsequently, total RNA was quantified by Agilent 2100 bioanalyzer (Thermo Fisher Scientific, MA, USA). Library was prepared with 1 μg of total RNA for each sample. Total RNA was purified by PAGE electrophoretic separation and the 18- to 30-t small RNA bands were excised and recovered. After being ligated to a 5’-adaptor and a 3’-adaptor, reverse transcription into cDNA was conducted by SuperScript II Reverse Transcriptase (Invitrogen, USA), and the cDNA fragments were further enriched by PCR amplification. The PCR products (100–120 bp) were selected and purified by QIAquick Gel Extraction Kit (QIAGEN, Valencia, CA). After measuring the quality and quantitation of library, the PCR products were sequenced on the Illumina Hiseq platform (Illumina, San Diego, CA, USA).

### Identification of DEMs

The raw data from miRNA sequencing were subjected to data filtering for obtaining clean reads. The known miRNAs were identified by comparing the sequencing tags with the miRBase database using blast software. Moreover, the novel miRNAs were predicted by mapping the clean reads to the human reference genome using the Mireap software. The target genes of miRNAs were predicted by the TargetScan (18) database. The DEMs between CHOL and control samples were analyzed using the DEseq2 package (19) with the cutoff value of |log2 fold change (FC)| > 1 and p < 0.05. The volcano plot of DEMs was created to visualize the DEMs.

### Functional enrichment analysis of target genes of DEMs

To understand the function of DEMs, Gene Ontology (GO) and Kyoto Encyclopedia of Genes and Genomes (KEGG) pathway enrichment analyses for target genes of DEMs were conducted using the phyper function in R. The p-value was adjusted as false discovery rate (FDR), and the significantly enriched GO terms or pathways were obtained with FDR ≤ 0.01.

### Real-time quantitative polymerase chain reaction

The expression levels of candidate serum exosomal miRNAs were validated in serum exosomes from another 10 healthy individuals and 10 patients with CHOL (4 intrahepatic and 6 extrahepatic) using qPCR. In detail, total RNA was extracted from serum exosomes using Trizol reagent (CW Bio Co., Ltd., Beijing, China). Subsequently, reverse transcription into cDNA was carried out using the HiFiScript cDNA Synthesis kit (CW Bio Co., Ltd.). Real-time qPCR was then completed using specific primers (**Table 1**) and the SYBR Master Mixture Taq kit (CW Bio Co., Ltd.). U6 was used as the reference.

**Table 1:** The primers used in this study.

Genes	Forward	Reverse
U6	GTGCTCGCTTCGGCAGCACATAT	AGTGCAGGGTCCGAGGTATT
miR-1292-5p	GGAACGGGTTCCGGCAG	AGTGCAGGGTCCGAGGTATT
miR-342-5p	CGCGAGGGGTGCTATCTGT	AGTGCAGGGTCCGAGGTATT
miR-4732-5p	GCGTGTAGAGCAGGGAGCAG	AGTGCAGGGTCCGAGGTATT
miR-3124-5p	ATTCGCGGGCGAAGGC	AGTGCAGGGTCCGAGGTATT
miR-3115	CGCGCGATATGGGTTTACTA	AGTGCAGGGTCCGAGGTATT

### Cell culture

Human CHOL cell lines HuCCT1 (JCRB, Osaka, Japan) and RBE (Institute of Biochemistry and Cell Biology, Shanghai Institutes for Biological Sciences, Chinese Academy of Sciences, Shanghai, China) were cultured in Dulbecco’s modified Eagle’s medium (DMEM, Gibco, USA) containing 10% exosome-depleted fetal bovine serum (FBS, Gibco, USA), and maintained at 37°C in a 5% CO2 atmosphere.

### qPCR

Exosomes were isolated from HuCCT1 and RBE cells by the hypervelocity centrifugation method. Then, qPCR was also performed to determine the expression levels of candidate serum exosomal miRNAs in HuCCT1 and RBE cells. The PCR method was conducted as described above.

### Cell transfection and treatment

The lentivirus for miR-3124-5p overexpression (Lv-OE-miR-3124-5p), miR-3124-5p knockdown (Lv-KD-miR-3124-5p), and their negative controls (Lv-OE-NC or Lv-KD-NC) were obtained commercially from GenePharma (Shanghai, China). RBE cells (1 × 105) were seeded into each well of a six-well plate at 60%–80% confluence. Cells in different transfection groups, named OE-NC-Exo, OE-miR-3124-5p-Exo, KD-NC-Exo, and KD-miR-3124-5p-Exo, were transfected with Lv-OE-NC, Lv-OE-miR-3124-5p, Lv-KD-NC, and Lv-KD-miR-3124-5p at an appropriate MOI (multiplicity of infection). Transfection was performed using Lipofectamine 2000 (Invitrogen, Carlsbad, CA). After cells stuck to the wall and reached 50%–60% confluence, cells were washed twice in PBS and grown in serum-free medium for 48 h. Supernatants of different transfection groups were collected and the transfection efficiency was detected using qPCR. Using the hypervelocity centrifugation method, exosomes were extracted from different transfection groups. The exosomes in different treatment groups were labeled with the green fluorescent dye PKH67 (Sigma-Aldrich Co., St Louis, MO, USA). The PKH67-labeled exosomes were diluted in 500 μl of 2% complete culture medium and filtered by a 0.22-μm membrane for sterilization.

HUVECs were seeded in 24-well plates. After reaching 60% confluence, HUVECs were co-cultured with the PKH67-labeled exosomes for different time points (0, 4, 8, and 16 h). Cells were fixed with paraformaldehyde for 15 min. Then, cytoskeletons of HUVECs were stained with phalloidin (red) and nuclei were stained with DAPI (blue). After washing, 0.5 ml of PBS was added, and whether exosomes could enter HUVECs was observed by a fluorescence microscope (Olympus, Tokyo, Japan).

To further determine the function of exosomal miR-3124-5p in endothelial cells, HUVECs were co-cultured with the exosomes from RBE cells that were treated with PBS or transfected with Lv-OE-NC, Lv-OE-miR-3124-5p, Lv-KD-NC, and Lv-KD-miR-3124-5p. The concentration of exosomes acting on HUVECs was 50 μg/ml. After 48 h of treatment, cells were harvested for subsequent cell function detection.

### Transwell migration assay

The Transwell inserts (8-μm pore size, Corning, USA) were added into a 24-well plate. Then, 100 μl of serum-free media was added into inserts and continued to incubate for 1–2 h. The medium was removed and 600 μl of medium containing 30% FBS was then added into the lower chamber of Transwell inserts. Cells at the logarithmic growth stage in different groups were digested with trypsin and suspended in serum-free media. The cell suspension was seeded into the upper chamber of Transwell inserts. After incubation for 24 h, the medium was removed and cells in the upper Transwell inserts were wiped with a cotton swab. The Transwell inserts were stained with 0.1% crystal violet for 20 min, then rinsed and air-dried. The Transwell inserts were imaged using a fluorescent microscope (Olympus) and the number of migrated cells was counted by ImageJ software (National Institutes of Health).

### CCK-8 assay

Cells at the logarithmic growth stage in different treatment groups were digested with trypsin and suspended in complete medium. Cells (2,000) were seeded onto 96-well plates. At the indicated time points (0, 24, 48, 72, and 96 h), 10  μl of CCK-8 solution (5 mg/ml, Med Chem Express, NJ, USA) was added to each well. After incubating the cells for 1–4 h at 37°C, the optical density (OD) value was measured with a microplate reader (Tecan Infinite F50, Tecan, Männedorf, Switzerland).

### EdU (5-Ethynyl-2’-deoxyuridine) assay

Cell proliferation was evaluated using the kFluor488 Click-iT EdU Kit (KeyGEN, Nanjing, China) according to the manufacturer’s instructions. Cells were seeded into 12-well plates. At 48 h of different treatments, 250 μl of preheated 2 × EdU (20 μmol/L) and 250 μl of medium were added, and the cells continued to incubate for 24 h at 37°C in a 5% CO2 atmosphere. Cells were fixed in 4% paraformaldehyde for 30 min at room temperature, incubated with PBS containing glycine (2 mg/ml) for 5 min, washed three times with PBS containing 3%BSA, and treated with 0.2% Triton X-100 for 20 min. Then, 500 μl of Click-iT mixture was added and continued to incubate cells for 30 min at room temperature away from light. Cellular nuclei were counterstained with Hoechst 33342 (5 μg/ml) for 15–30 min at room temperature away from light. The positive fluorescent signals were observed with an inverted fluorescence microscope (Olympus). The proportion of EdU-positive cells was calculated as (EdU-stained cells/Hoechst-stained cells) ×100%.

### Scratch wound healing assay

The lines were drawn on the back of a six-well plate with a marker pen. Cells (5 × 105 cells/well) were plated in each well of a six-well plate. After overnight culture, a confluent cell monolayer was formed. Subsequently, we created a linear scratch wound with a pipette tip. Scratched wells were rinsed with PBS to remove the debris, and the serum-free media were added. Cells were then maintained in a 37°C incubator with 5% CO2 for 48 h. At 0 and 48 h after scratch, wound closure was photographed and the relative wound healing area was analyzed by ImageJ software.

### Tube formation assay

HUVECs were seeded into a six-well plate. After complete adherence, HUVECs were incubated with PBS or exosomes isolated from different transfected RBE cells for 48 h. Afterwards, HUVECs (5 × 105 cells/ml) were collected and resuspended with complete ECM medium. Then, 500  μl of cell suspension was seeded into a 24-well plate coated with the polymerized Matrigel (BD Biosciences, San Jose, CA, USA). After incubation at 37°C for 24 h, the images of capillary-like structures were scanned using a light microscope (40×, Olympus). The blood vessel area, total length, junction count, and node count were analyzed by ImageJ software.

### Dual-luciferase reporter gene assay

The wild or mutated GDF11-3’UTR was cloned into the psiCHECKTM-2 dual luciferase reporter plasmid (Promega, Madison, Wisconsin, USA), named psiCHECKTM-2-GDF11-WT/MUT. Cells (2 × 104 cells/well) were cultured in 96-well plates and cotransfected with the psiCHECKTM-2-GDF11-WT/MUT and miR-3124-5p mimics or mimic NC using Lipofectamine 2000 (Invitrogen). At 48 h after transfection, the cells were lysed to detect luciferase activity on the dual-Luciferase Reporter Assay System (E1910, Promega). Renilla luciferase activity was standardized to firefly activity, and the relative luciferase activity was calculated.

### Western blot assay

Exosomes and cells were lysed with 1 × RIPA lysis buffer (Beyotime, Haimen, China) on ice for 10–15 min. After centrifugation at 12,000 ×g for 5 min at 4°C, total protein extracts were collected and their concentration was detected using the BCA method. The protein (20 μg) was then separated on 10% SDS-polyacrylamide gels and the blots were transferred onto polyvinylidene fluoride (PVDF) membranes (Millipore, Billerica, MA, USA). After blocking with 5% skimmed milk, the membranes were then incubated with primary antibodies at 4°C overnight and secondary antibodies at room temperature for 1 h. The primary antibodies to HSP70, CD81, TSG101, GDF11, and GAPDH (1:2,000, Proteintech, Inc., CA, USA) and corresponding secondary antibodies (anti-rabbit or anti-mouse IgG, 1:5,000, ABclonal, Cambridge, MA, USA) were used. After rinsing, the blots were visualized using the ECL method (Pierce, Thermo Scientific, Waltham, USA) and the protein signals were analyzed using ImageJ software.

### Tumor xenograft models

All animal experiments were approved by the Animal Care Committee of Shandong University. A total of 25 female 4-week-old nude mice were obtained from Beijing Vital River Laboratory Animal Technology Co., Ltd. (Beijing, China). RBE cells at logarithmic growth phase were centrifuged at 1,500 rpm for 10 min and washed three times with sterile normal saline. RBE cells were suspended in normal saline, and the concentration of cell suspension was adjusted as 1 × 107 cells/ml. Then, 100 µl of cell suspension was injected subcutaneously into the right axilla of mice to establish the xenograft model. When the subcutaneous tumor volume reached about 80–100 mm3, mice were randomly divided into five groups (n = 5 each group) and treated with PBS, OE-NC-Exo, OE-miR-3124-5p-Exo, KD-NC-Exo, and KD-miR-3124-5p-Exo at multiple points every other day for 15 days. The body weight and tumor volume were recorded every 4 days. The mice were kept for 1 week and were sacrificed with cervical dislocation. The tumor was completely stripped and its weight and volume were measured.

### Statistical analysis

All experiments were repeated more than three times independently. Statistical analyses were accomplished by the SPSS 18.0 software (SPSS, Chicago, IL). Data are expressed as the mean ± standard deviation (SD). Data conforming to normal distribution were compared with ANOVA and Tukey’s post-hoc test. Otherwise, data were analyzed by non-parametric test. P < 0.05 was deemed statistically significant.

## Results

### Characterization of exosomes from the serum

**Figure 1:**
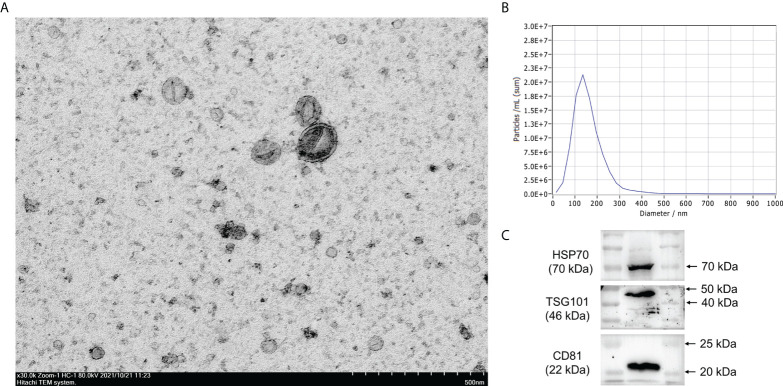
Characterization of exosomes. **(A)** Exosomes were extracted from human serum and morphological characterization was observed by transmission electron microscopy. **(B)** The size of exosomes was analyzed by nanoparticle tracking analysis (NTA). **(C)** Exosome protein markers HSP70, CD81, and TSG101 were identified by Western blot analysis.

The cup-like exosome particles were observed by TEM, suggesting that exosomes were successfully isolated (**Figure 1A**). NTA revealed that most of the particles had a size of <200 nm (peak: 152.3 nm) diameter (**Figure 1B**), which were morphologically consistent with the exosomes. Western blot analysis further showed that these vesicles expressed the exosomal markers HSP70, CD81, and TSG101 (**Figure 1C**).

### Identification of exosomal DEMs between CHOL and control samples

**Figure 2:**
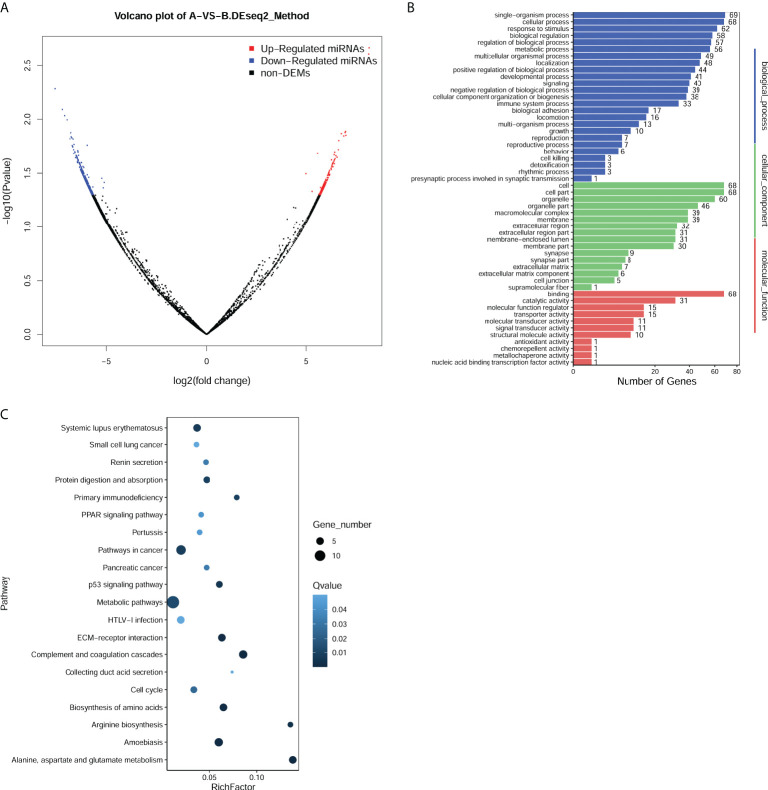
Identification and function enrichment analysis of differentially expressed miRNAs (DEMs) in exosomes from cholangiocarcinoma (CHOL) compared to healthy controls. **(A)** Volcano plot of exosomal DEMs between CHOL and control samples. **(B)** Gene Ontology (GO) enrichment analyses for target genes of DEMs. **(C)** Kyoto Encyclopedia of Genes and Genomes **(KEGG)** pathway enrichment analyses for target genes of DEMs.

miRNA sequencing was performed to determine miRNA expression profiles in exosomes derived from serum of CHOL patients or healthy controls. With the cutoff value of |log2 FC| >1 and p < 0.05, a total of 632 exosomal DEMs were identified between CHOL and control samples, including 190 upregulated and 442 downregulated miRNAs (**Figure 2A**). Based on the fold change, the top 10 upregulated miRNAs were has-miR-1292-5hashsa-miR-342-5p, hsa-miR-1255hasp, hsa-miR-4732-5p, hsa-miR-3124-5p, novel-m8096-5p, hsa-miR-3115, novel-m2798-3p, novel-m5911-5p, and novel-m11641-3p (**Table 2**). Functional enrichment analysis for target genes was then carried out. We found that the target genes of DEMs were significantly enriched in GO functions, such as biological process–cellular process, cellular component–cell part, and molecular function–binding (**Figure 2B**). Moreover, the target genes of DEMs were remarkably enriched in KEGG pathways, such as metabolic pathways, p53 signaling pathway, and complement and coagulation cascades (**Figure 2C**).

**Table 2:** The top 10 upregulated miRNAs between cholangiocarcinoma and control samples.

miRNA_name	log2 fold change	*p*-value
hsa-miR-1292-5p	8.143825	0.002171
hsa-miR-342-5p	8.12519	0.002484
hsa-miR-1255b-5p	6.975075	0.014051
hsa-miR-4732-5p	6.95696	0.012924
hsa-miR-3124-5p	6.932139	0.01324
novel-m8096-5p	6.867978	0.014263
hsa-miR-3115	6.744624	0.013553
novel-m2798-3p	6.619973	0.015715
novel-m5911-5p	6.599649	0.01912
novel-m11641-3p	6.534037	0.022501

### Exosomal miR-3124-5p was selected as a key player in CHOL development

**Figure 3:**
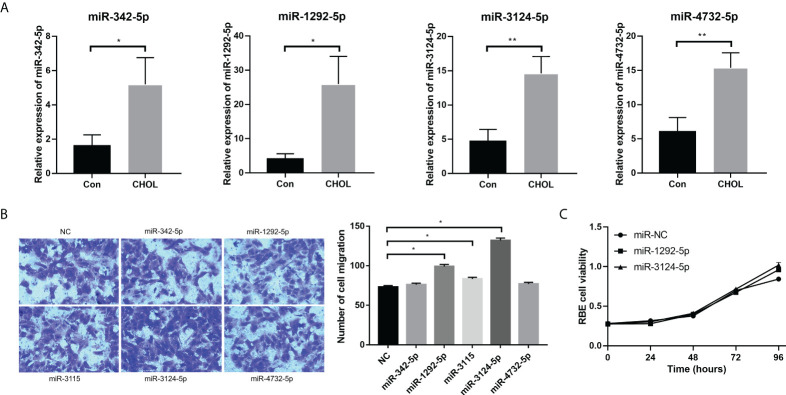
The effects of overexpression of exosomal miRNAs (miR-1292-5p, miR-342-5p, miR-4732-5p, miR-3124-5p, and miR-3115) on the migration and proliferation of RBE cells. **(A)** qPCR showed the expression of miR-342-5p, miR-1292-5p, miR-3124-5p, and miR-4732-5p in serum exosomes from healthy individuals and patients with CHOL. **(B)** Transwell assay showed the effect of overexpression of exosomal miRNAs on RBE cell migration. **(C)** CCK-8 assay showed the effect of overexpression of exosomal miRNAs on RBE cell viability. Compared to NC, *p < 0.05 and **p < 0.01.

Five upregulated exosomal miRNAs (miR-1292-5p, miR-342-5p, miR-4732-5p, miR-3124-5p, and miR-3115) with the largest differences in expression were selected for miRNA validation. We first determined the expression of these five miRNAs in serum exosomes from 10 healthy individuals and 10 patients with CHOL by qPCR. The subject characteristics are shown in **Supplementary Table 1**. Consistent results were obtained: miR-1292-5p, miR-342-5p, miR-4732-5p, and miR-3124-5p were significantly upregulated in serum exosomes from patients with CHOL compared to those in serum exosomes from healthy controls (**Figure 3A**). However, the expression of miR-3115 in serum exosomes was too low to be detected.

We further conducted qPCR to detect the background expression levels of five miRNAs in the exosomes from HUCCT1 and RBE cells. The results showed that only the background expression of miR-3124-5p in HUCCT1 exosomes and the background expression of miR-4732-5p and miR-3124-5 in RBE exosomes were relatively ideal, and the background expression of other miRNAs were very low (**Supplementary Table 1**). Moreover, RBE cells were transfected with miR-1292-5p mimics, miR-342-5p mimics, miR-4732-5p mimics, miR-3124-5p mimics, and miR-3115 mimics for overexpression of these miRNAs. Transwell migration assay revealed that the number of migrated RBE cells was significantly increased after overexpression of miR-1292-5p, miR-3124-5p, or miR-3115 (**Figure 3B**). CCK-8 assay showed that RBE cell viability was remarkably promoted after overexpression of miR-1292-5p or miR-3124-5p (**Figure 3C**). Notably, the effects of overexpression of miR-3124-5p on cell migration were stronger than overexpression of miR-1292-5p; thus, miR-3124-5p was selected for subsequent experiments.

### Exosomal miR-3124-5p promoted the proliferation, migration, and angiogenesis of endothelial cells

**Figure 4:**
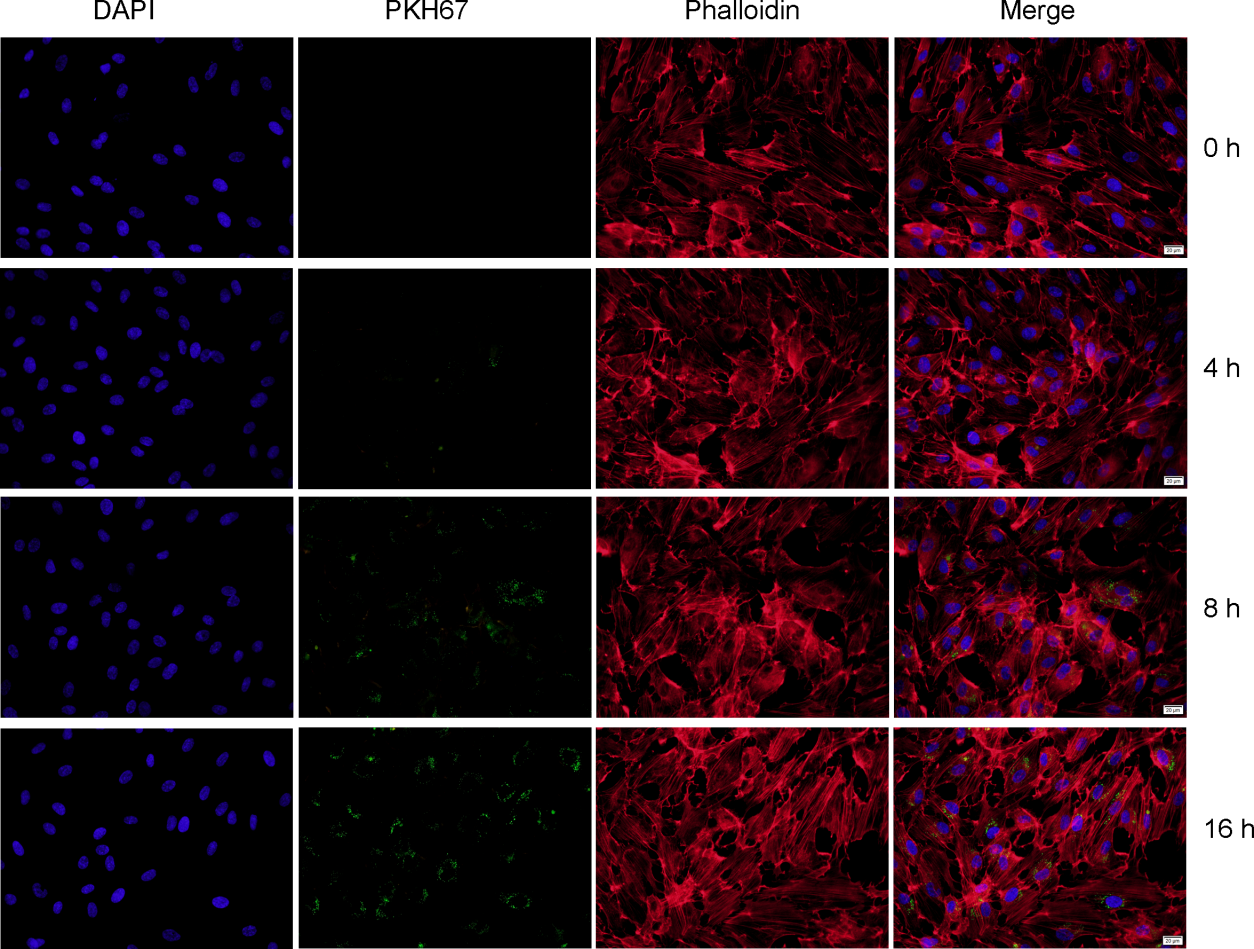
Fluorescence staining showed that exosomes from the supernatant of RBE cells could enter HUVECs. Nuclei and cytoskeleton of HUVECs were labeled with DAPI (blue) and phalloidin (red), respectively. Exosomes were labeled with PKH-67 (green). PKH67-labeled exosomes (green) were incubated with HUVECs, and PKH67-labeled exosomes were detected inside the HUVECs as visualized using a fluorescence microscope, indicating that the exosomes entered into the HUVECs.

**Figure 5:**
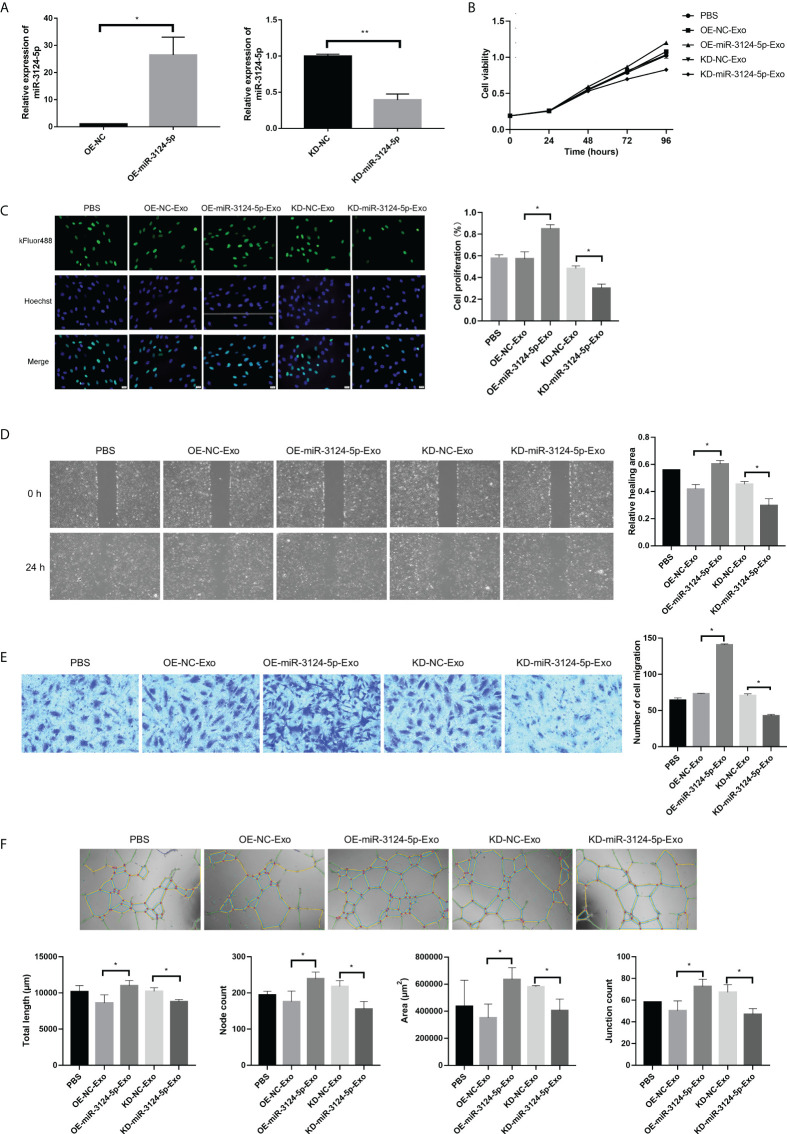
The effects of exosomes from RBE cells with aberrant miR-3124-5p expression on the proliferation, migration, and angiogenesis of endothelial cells. HUVECs were co-cultured with the exosomes in different treatment groups (PBS, OE-NC-Exo, OE-miR-3124-5p-Exo, KD-NC-Exo, and KD-miR-3124-5p-Exo). **(A)** qPCR showed the expression of miR-3124-5p in RBE cells after transfection. **(B)** CCK-8 assay showed cell viability of HUVECs in different treatment groups. **(C)** EdU assay showed HUVEC proliferation in different treatment groups. **(D)** Scratch wound healing assay showed the healing abilities of HUVECs in different treatment groups. **(E)** Transwell migration assays indicated HUVEC migration in different treatment groups. **(F)** Tube formation assay revealed the total length, node count, area, and junction count of HUVECs in different treatment groups. *p < 0.05. and **p < 0.01

To further determine the function of exosomal miR-3124-5p, HUVECs were firstly co-cultured with the PKH67-labeled exosomes for different time points. It was observed under fluorescence microscope that PKH67-labeled (green) exosomes were present in HUVECs (red and blue) using fluorescence microscopy, demonstrating that exosomes from the supernatant of RBE cells could enter HUVECs (**Figure 4**). Then, we overexpressed and knocked down the expression of miR-3124-5p in RBE cells by transfection. HUVECs were co-cultured with the exosomes in different treatment groups (PBS, OE-NC-Exo, OE-miR-3124-5p-Exo, KD-NC-Exo, and KD-miR-3124-5p-Exo), and the effects of exosomes from RBE cells with aberrant miR-3124-5p expression on the proliferation, migration, and angiogenesis of endothelial cells were analyzed. As shown in **Figure 5A**, miR-3124-5p was successfully overexpressed and knocked down in RBE cells (p < 0.05). The results of both CCK-8 (**Figure 5B**) and EdU (**Figure 5C**) assays showed that exosomes from miR-3124-5p overexpression RBE cells significantly promoted HUVEC proliferation, whereas exosomes from miR-3124-5p knockdown RBE cells had the opposite effect (p < 0.05). The results of both scratch wound healing (**Figure 5D**) and Transwell migration (**Figure 5E**) assays indicated that HUVEC migration was significantly enhanced after co-culture with exosomes from miR-3124-5p-overexpressing RBE cells, but remarkably inhibited after co-culture with exosomes from miR-3124-5p knockdown RBE cells (p < 0.05). The results of tube formation assay revealed that the total length, node count, area, and junction count were all significantly increased after the addition of exosomes from miR-3124-5p overexpression, suggesting that exosomes from miR-3124-5p overexpression RBE cells enhanced tube formation in HUVECs; however, exosomes from miR-3124-5p knockdown RBE cells had the opposite effect (p < 0.05, **Figure 5F**).

### miR-3124-5p downregulated GDF11 expression

**Figure 6:**
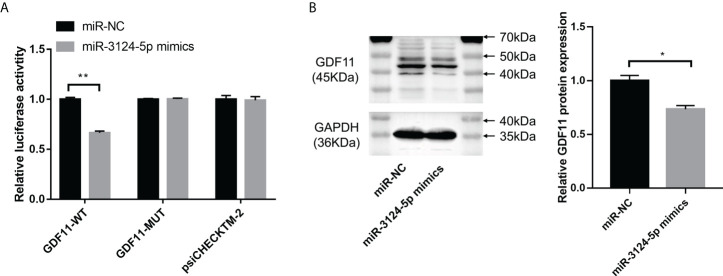
miR-3124-5p targeted GDF11 and downregulated GDF11 expression. **(A)** Luciferase activity assay confirmed that miR-3124-5p could target GDF11-WT. **(B)** Western blot assay showed GDF11 protein expression in miR-3124-5p mimic group and miR-NC group. Compared to miR-NC group, *p < 0.05, **p < 0.01.

To explore the mechanism of miR-3124-5p in regulating tumor metastasis and angiogenesis, we predicted the target genes of miR-3124-5p in CHOL by the TargetScan database. Among target genes, GDF11 has been shown to play a key role in diverse diseases, such as cardiovascular disease (20), acute pancreatitis (21), and colorectal cancer (22). However, the function of GDF11 in CHOL is largely unknown. We thus investigated the target relationship between miR-3124-5p and GDF11. Luciferase activity assay based on luciferase constructs containing GDF11-WT and GDF11-MUT showed a significant inhibition of luciferase activity of GDF11-WT by co-transfection with miR-3124-5p (p < 0.01), while little effect of it on GDF11-WT (**Figure 6A**). Moreover, the results of Western blot assay showed that the expression of GDF11 protein in the miR-3124-5p mimics group were significantly lower than that in the miR-NC group (p < 0.05, **Figure 6B**).

### Exosomal miR-3124-5p promoted tumor growth

**Figure 7:**
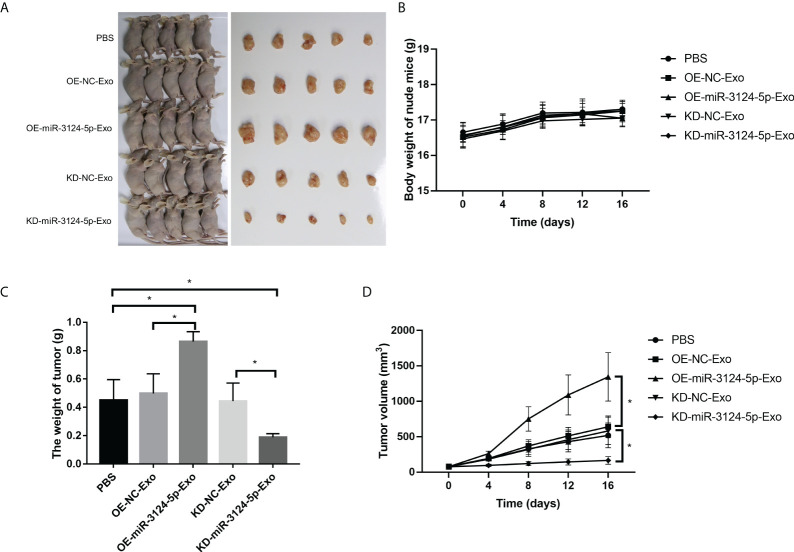
Exosomal miR-3124-5p promoted tumor growth *in vivo*. **(A)** Photographs of macroscopic tumor appearance. **(B)** Body weight of nude mice of different groups. **(C)** Tumor weight of different groups. **(D)** Tumor volume of different groups. *p < 0.05.

We further investigated the effects of exosomal miR-3124-5p on tumor growth in vivo (**Figure 7A**). The results showed that there was no significant effect of different treatments on body weight of nude mice (**Figure 7B**). However, tumor weight (**Figure 7C**) and tumor volume (**Figure 7D**) were significantly increased after treatment with OE-miR-3124-5p-Exo and remarkably decreased after treatment with KD-miR-3124-5p-Exo (p < 0.05). These data confirmed that overexpression of exosomal miR-3124-5p promoted tumor growth in vivo.

## Discussion

CHOL is a deadly cancer worldwide with limited available therapies. It would be valuable to explore a more sensitive biomarker for the early diagnosis of CHOL and for the prediction of disease metastases. miRNAs are revealed as important regulators in cancers, which may modulate angiogenesis, cell cycle progression, metastasis, apoptosis, autophagy, and therapy resistance (23). Several miRNAs have been observed to be dysregulated in CHOL and are associated with CHOL-related risk factors (24). For instance, miR-1182 and let-7a exert a synergistic antitumor effect on the development of CHOL in vitro and in vivo (25). Elevated miR-99b expression is found to be associated with the poorer overall survival of CHOL patients (26, 27). Circulating miRNA-150 is considered as a non-invasive, sensitive serum biomarker for CHOL diagnosis (28). Therefore, identification of crucial miRNAs will enrich our understanding of CHOL pathogenesis.

In recent years, miRNAs in exosomes has attracted increasing attention. Exosomes play an important role in cancer progression and have been widely used as biomarkers or therapeutic targets in the field of oncology (29, 30). Exosomes can effectively load and deliver miRNAs, leading to partial silencing of some genes in recipient cells, which, in turn, affects the activities of metastasis-promoting tumor cells (31). Growing evidence indicates that tumor cells communicate with other tumor cells or cells present in the tumor microenvironment through secretion and transfer of exosomal miRNAs (32). Herein, we investigated the expression profiles of miRNAs in serum exosomes of patients with CHOL using miRNA sequencing. The results showed that 632 exosomal DEMs were identified between CHOL and control samples, suggesting that these DEMs may be associated with CHOL. Furthermore, functional enrichment analysis further revealed that the target genes of DEMs were significantly enriched in pathways, such as the p53 signaling pathway. A previous study has revealed that miR-146b-5p inhibits CHOL development through modulating p53 translocation to mitochondria (33). miR-191 promotes the development of intrahepatic CHOL by modulating the TET1-p53 pathway (34). These data suggest the crucial role of the p53 signaling pathway in CHOL development. Based on our results, we speculated that these exosomal DEMs may affect CHOL development via modulating the p53 signaling pathway.

Notably, exosomal miR-3124-5p was selected as a key player in CHOL development based on our experimental validations. A previous study has revealed that the level of miR-3124-5p in patients with thromboangiitis obliterans is significantly higher than that in healthy individuals (35), suggesting the potential role of miR-3124-5p in thromboangiitis obliterans. Moreover, miR-3124-5p has been shown to be associated with multiple cancers, such as gastric cardia adenocarcinoma (36) and triple-negative breast cancer (37). Despite these, the role and regulatory mechanism of miR-3124-5p in cancers have not been fully clarified. In this study, we found that miR-3124-5p was upregulated in the serum exosomes of patients with CHOL. Moreover, overexpression of miR-3124-5p significantly promoted cell migration and viability of RBE cells. Furthermore, overexpression of exosomal miR-3124-5p promoted the proliferation, migration, and angiogenesis of HUVECs, whereas knockdown of miR-3124-5p had the opposite effect. These data confirm that exosomal miR-3124-5p may contribute to the development of CHOL and serve as a promising biomarker for disease diagnosis and prognosis.

Exosomal miRNAs secreted by cancer cells can be transferred to recipient cells to regulate gene expression (31, 38). Exosomal miR-23a can increase angiogenesis in gastric cancer via targeting Phosphatase and Tensin Homolog (PTEN) (39). Exosomal miR-141 is shown to promote angiogenesis and malignant progression of lung cancer through targeting growth arrest-specific homeobox gene (GAX) (40). In this study, we investigated the target relationship between miR-3124-5p and GDF11 to elucidate the possible mechanism of exosomal miR-3124-5p in CHOL. Growth differentiation factor 11 (GDF11) is a secreted protein in the TGF-β superfamily and the BMP subfamily and exerts tumor-suppressive function in a variety of many cancers, such as triple-negative breast cancer (41), pancreatic cancer (42), and hepatocellular carcinoma (43). Although the role of GDF11 in CHOL has not been investigated, our results suggested that GDF11 may be a key player in CHOL. Moreover, our study revealed that miR-3124-5 could directly target GDF11. It can therefore be speculated that exosomal miR-3124-5p may promote the development of CHOL via downregulating GDF11.

However, our study also has some limitations. Firstly, the sample size for miRNA sequencing was small. Secondly, the expression of GDF11 in tumors isolated from animal models was not detected. Lastly, we did perform in-depth mechanism studies to elucidate the regulatory mechanism of exosomal DEMs in CHOL development. More studies are still warranted to confirm our findings.

In conclusion, our study for the first time revealed the important function of exosomal miR-3124-5p and GDF11 in CHOL development. miR-3124-5p could enter HUVECs via the exosome pathway, and exosome-encapsulated miR-3124-5p promoted the malignant progression of CHOL by targeting the expression of GDF11. Exosomal miR-3124-5p and GDF11 could be promising biomarkers or therapeutic targets for the diagnosis and treatment of CHOL.

## Data availability statement

The datasets presented in this study can be found in online repositories. The names of the repository/repositories and accession number(s) can be found in the article/**Supplementary Material**.

## Ethics statement

This study was reviewed and approved by Ethics Committee of Qilu Hospital of Shandong University. The patients/participants provided their written informed consent to participate in this study. The animal study was reviewed and approved by the Animal Care Committee of Shandong University.

## Author contributions

HG: Methodology, Investigation, Data curation, and Writing—Original draft preparation. ZH: Investigation and Software. CG: Data curation and Investigation. NL: Investigation. ZZ: Visualization and Investigation. WN: Software and Validation. JN: Supervision. CP: Conceptualization, Funding acquisition, and Writing—Reviewing and Editing. All authors contributed to the article and approved the submitted version.

## Funding

This work was supported by the National Natural Science Foundation of China under Grant number 82072647, and the Natural Science Foundation of Shandong Province under Grant number ZR2020MH258.

## Conflict of interest

The authors declare that the research was conducted in the absence of any commercial or financial relationships that could be construed as a potential conflict of interest.

## Publisher’s note

All claims expressed in this article are solely those of the authors and do not necessarily represent those of their affiliated organizations, or those of the publisher, the editors and the reviewers. Any product that may be evaluated in this article, or claim that may be made by its manufacturer, is not guaranteed or endorsed by the publisher.
