# Supplementary material for: Exosome-transmitted miR-3124-5p promotes cholangiocarcinoma development via targeting GDF11
Source: Front Oncol. 2022 Aug 1;12:936507. doi: 10.3389/fonc.2022.936507 (PMC9376483; doi:10.3389/fonc.2022.936507)
Supplement: Supplementary file 1 [file Table_1.docx]

Supplementary Table 1 Subject characteristics

| Characteristics | CHOL patients  (n = 10) | Healthy controls (n = 10) |
| --- | --- | --- |
| Age (years) | 63.4 ± 11.7 | 53.2 ± 19.1 |
| Male | 8 | 3 |
| Maximal tumor size(cm) | 2.2 ± 1.1 | NA |
| Tumor stage |  |  |
| I-II | 9 | NA |
| III-IV | 1 | NA |
| Differentiation |  |  |
| High | 3 | NA |
| High-Moderate | 3 | NA |
| Moderate | 2 | NA |
| Moderate-Low | 2 | NA |
| Vascular invasion | 5 | NA |
| Location |  |  |
| Intrahepatic | 4 | NA |
| Extrahepatic | 6 | NA |
| HBV | 0 | 1 |
| HCV | 0 | 0 |
| Biliary parasite | 0 | 0 |
| Biliary stone | 3 | 7 |
| Diabetes | 2 | 2 |
